# Mutation of the galectin‐3 glycan‐binding domain ( *Lgals3‐*R200S) enhances cortical bone expansion in male mice and trabecular bone mass in female mice

**DOI:** 10.1002/2211-5463.13483

**Published:** 2022-09-14

**Authors:** Kevin A. Maupin, Cassandra R. Diegel, Payton D. Stevens, Daniel Dick, VAI Vivarium and Transgenic Core, Bart O. Williams

**Affiliations:** ^1^ Van Andel Institute Grand Rapids MI USA

**Keywords:** bone μCT, CRISPR/Cas9, galectin, genetic animal models, sexual dimorphism

## Abstract

We previously observed that genomic loss of galectin‐3 (Gal‐3; encoded by *Lgals3*) in mice has a significant protective effect on age‐related bone loss. Gal‐3 has both intracellular and extracellular functionality, and we wanted to assess whether the affect we observed in the *Lgals3* knockout (KO) mice could be attributed to the ability of Gal‐3 to bind glycoproteins. Mutation of a highly conserved arginine to a serine in human Gal‐3 (*LGALS3*‐R186S) blocks glycan binding and secretion. We generated mice with the equivalent mutation (*Lgals3*‐R200S) and observed a subsequent reduction in Gal‐3 secretion from mouse embryonic fibroblasts and in circulating blood. When examining bone structure in aged mice, we noticed some similarities to the *Lgals3*‐KO mice and some differences. First, we observed greater bone mass in *Lgals3*‐R200S mutant mice, as was previously observed in *Lgals3*‐KO mice. Like *Lgals3*‐KO mice, significantly increased trabecular bone mass was only observed in female *Lgals3*‐R200S mice. These results suggest that the greater bone mass observed is driven by the loss of extracellular Gal‐3 functionality. However, the results from our cortical bone expansion data showed a sex‐dependent difference, with only male *Lgals3*‐KO mice having an increased response, contrasting with our earlier study. These notable sex differences suggest a potential role for sex hormones, most likely androgen signaling, being involved. In summary, our results suggest that targeting extracellular Gal‐3 function may be a suitable treatment for age‐related loss of bone mass.

## Graphical abstract

Galectin‐3 (*Lgals3*) has glycan‐binding and protein–protein interactions. Knockout mice (*Lgals3*‐KO) have sexually dimorphic protection against bone loss. A mutation in the glycan‐binding domain (Human:R186S) disrupts galectin‐3 glycan‐binding and secretion *in vitro*. Generating the equivalent mutation in mice (R200S), we observed reduced galectin‐3 secretion and increased bone mass at 36 weeks, suggesting the importance of the glycan‐binding domain on the phenotype.

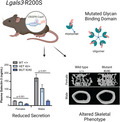

**Abbreviations**
aBMD: areal bone mineral density
B.Ar: bone area
BMD: bone mineral density
BV/TV: bone volume fraction
Conn.D: connectivity density
DEXA: dual emission X‐ray absorptiometry
ELISA: enzyme‐linked immunosorbent assays
Gal‐3: galectin‐3
HET: heterozygotes
KO: knockout
KI: knock‐in
M.Ar: marrow area
MEF: mouse embryonic fibroblast
μCT: micro‐computed tomography
MMI: mass moment of inertia
MUT: mutants
PAM: protospacer adjacent motif
%BF: percent bodyfat
ssODN: single‐stranded oligo donor
T.Ar: total area
Tb.N: trabecular number
Tb.Th: trabecular thickness
Tb.Sp: trabecular spacing
WT: wild‐types

Galectin‐3 (Gal‐3; encoded by *Lgals3*) is a protein that functions outside the cell to regulate glycoprotein secretion and turnover [1, 2] and intracellularly in protein chaperoning and mRNA splicing [3, 4]. We previously found that genomic deletion of Gal‐3 in mice (*Lgals3*‐KO) protects the mice against age‐related [5] or sex‐hormone deprivation bone loss [6]. A better understanding of the intracellular vs. extracellular functions of Gal‐3 could lead to more informed drug development to treat osteoporosis, fibrosis, and other pathologies [7, 8, 9, 10, 11, 12, 13]. Thus far, most functional studies of Gal‐3 have yielded conflicting results. Take, for example, the mechanism of how Gal‐3 regulates β‐catenin protein levels [14, 15, 16, 17, 18, 19, 20, 21, 22, 23, 24, 25, 26, 27]. Contradictory findings have suggested that the mechanism of action is either via a direct intracellular interaction [7, 9, 17, 24], or via an indirect extracellular interaction [20, 25, 28, 29].

In order to determine whether the extracellular function of Gal‐3 is necessary for its effect on age‐related bone loss, we generated a secretion deficient mouse knock in variant of Gal‐3. Gal‐3 can be mutated to selectively disrupt its glycan‐binding and secretion functions while preserving its intracellular functions by mutating a highly conserved arginine to a serine in the glycan‐binding domain of human Gal‐3 (R186S) [30]. The R186S mutation lacks extracellular function in *in vitro* assays and is unable to bind any serum glycoproteins [30, 31]. Mutation of the structurally equivalent arginine in galectin‐7 blocks glycan‐binding but still allows galectin‐7 to function intracellularly [32, 33]. Dissociation of intracellular interactions and regulation of mRNA splicing from extracellular glycan‐binding has also been described for galectin‐1 [34]. We created the equivalent R186S mutation in mouse Gal‐3 (*Lgals3*‐R200S) to prevent extracellular secretion of Gal‐3 and assessed the consequences of this mutation on age‐related bone loss *in vivo*.

## Materials and methods

### Experimental animals

B6C3F1/J mice were obtained from Jackson Labs (Bar Harbor, ME, USA) and were used to generate our B6;C3‐*Lgals3* ^*tm1Vari*^ mutant model, which will be referred to as *Lgals3‐*R200S.

Once this line was generated, we backcrossed it for two generations with purebred C57BL/6J mice. All animal procedures followed the protocol (PIL‐16‐01‐002) approved by the Institutional Animal Care and Use Committee of the Van Andel Institute. Mice were housed in climate‐controlled conditions (25 °C, 55% humidity, and 12 h of light alternating with 12 h of darkness) and fed a standard LabDiet Rodent Chow 5010 (Purina Mills, Gray Summit, MO, USA). Animals for our studies were euthanized at embryonic day 13 to collect mouse embryonic fibroblasts (MEFs) or at a 36‐week time point for bone studies.

### gRNA design and synthesis

Guides were designed to encompass 40 bp upstream and downstream of the target mutation site (*Lgals3* codon 200 in exon 5) using the Zhang Lab's Optimized CRISPR Design Tool (crispr.mit.edu). A potential sgRNA binding site with a protospacer adjacent motif (PAM; AGG) was identified 8 bp downstream from codon 200. This sgRNA was selected for its proximity to the desired mutation site and low predicated probability for off‐target binding (quality score 65).

A 20 bp DNA fragment (synthesized by Integrated DNA Technologies (IDT), San Diego, CA, USA) representing the sgRNA plus BbsI complementary overhangs (sgRNA FWD: 5’‐CACCTTTGCCACTCTCAAAGGGGA‐3′ and sgRNA REV: 5′‐AAACTCCCCTTTGAGAGTGGCAAA‐3′) was cloned into a BbsI digested pX330‐U6‐Chimeric_BB‐CBh‐hSpCas9 plasmid (42230; Addgene, Cambridge, MA, USA). Template DNA for *in vitro* transcription was generated by PCR amplification of the gRNA sequence using Phusion HF DNA polymerase and a primer set (synthesized by IDT) consisting of a FWD primer recognizing the cloned sgRNA sequence with a T7 RNA polymerase recognition sequence fused on the 5′ end (5’‐AATACGACTCACTATAGGGTTTGCCACTCTCAAAGGGGA‐3′) and a REV primer that recognized the terminal end of the gRNA scaffold sequence on the pX300 plasmid (5’‐AAAAGCACCGACTCGGTGCC‐3′). After verifying consistent production of a single product by agarose gel electrophoresis, the template was purified using the QIAquick PCR purification kit (Qiagen, Germantown, MD, USA) and eluted with RNAse free water.

*In vitro* transcription was performed on the purified gRNA template using a MEGAshortscript Kit (Ambion; Thermo Fisher Scientific, Waltham, MA, USA). The synthesized RNAs were pooled, treated with DNase to remove the remaining template, and purified using a MEGAclear Kit (Ambion; Thermo Fisher Scientific). The purity of eluted gRNA product was determined by formalin gel electrophoresis, and gRNA was quantified on a Nanodrop 2000c (Thermo Fisher Scientific). gRNA was stored at −80 °C.

### Single strand oligonucleotide donor template

The single stranded oligonucleotide (ssODN) template for homologous recombination (200 bases, PAGE purified) was synthesized by IDT. The sequence was: 5′‐ATGATGTTGCCTTCCACTTTAACCCCCGCTTCAATGAGA ACAACAGGAGAGTCATTGTGTGTAACACGAAGCAGGACAATAACTGGGGAAAGGAAGAGTCACAATCTGCGTTCCCCTTTGAGAGTGGCAAACCATTCAAAGTAAGTTGGGGCTTTGGCTGTATGCGCACAGCGTTCTCTTACCAAGGGGAATCACGGAAA‐3′. Targeted mismatches are underlined in the ssODN sequence.

### Microinjection of mice

Mouse zygotes were obtained by mating superovulated B6C3F1/J females with B6C3F1/J males. RNAs and ssODN were thawed and mixed just prior to injections for final concentrations of 30 ng·μL^−1^ WT Cas9 mRNA (Sigma Aldrich, St. Louis, MO, USA), 15 ng·μL^−1^ gRNA, and 50 ng·μL^−1^ ssODN. The mix was microinjected into the pro‐nuclei of zygotes and transferred into pseudo‐pregnant females at the two‐cell stage. After identifying founders, we backcrossed the line to C57BL/6J twice before intercrossing to generate animals for our study.

### Genotyping

Genomic DNA was isolated from tail clips at weaning and necropsy by proteinase K digestion and ethanol precipitation. For the *Lgals3*‐R200S reaction, wild‐type mice (*Lgals3* ^+/+^) yielded a single 483 bp band, homozygous mutant mice (*Lgals3‐*R200S^KIKI^) yielded two 131 and 586 bp bands, and heterozygotes (*Lgals3‐*R200S^KI+^) had three bands of 131, 483, and 586 bp. Sanger sequencing verified that the correction mutations and no spurious indels were present in the *Lgals3‐*R200S allele. Tail tip DNA from *Lgals3‐*R200S‐positive animals was amplified by Phusion HF DNA polymerase PCR using primers encompassing exon 5 of *Lgals3* (FWD: 5′‐TTCAGGAGAGGGAATGATGTTG‐3′ and REV: 5’‐CTGAAGGAGCTGAAGGACAC‐3′). The product of this reaction was purified using a QIAquick PCR Purification Kit (Qiagen), then cloned into pMiniT Vectors with the NEB PCR Cloning Kit and transformed into NEB 10‐beta Competent *E. coli* (NEB, Ipswich, MA, USA). Colonies positive for the *Lgals3‐*R200S allele by PCR were grown overnight at 37 °C with nutation at 200 rpm. DNA from small aliquots of these cultures was then amplified using the TempliPhi DNA amplification kit and submitted to Genewiz (South Plainfield, NJ, USA) for Sanger sequencing using a forward primer upstream of the insertion site on the pMiniT Vector that came with the NEB PCR Cloning Kit. (5’‐ACCTGCCAACCAAAGCGAGAAC‐3′).

### Cell‐surface biotinylation of mouse embryonic fibroblasts

*Lgals3*‐R200S wild‐type, heterozygous and mutant MEFs were harvested from 13‐day mouse embryos. Cells were cultured on 10 cm dishes until they reached 70% confluency. Experiments were carried out at 0–4 °C to reduce biotin internalization. Cells were washed three times with ice‐cold PBS pH 8.0 and labeled with 1 mg·mL^−1^ EZ‐Link™ Sulfo‐NHS‐LC‐Biotin (Thermo Fisher Scientific) for 30 min with gentle rocking. The cells were then washed three times with ice‐cold PBS containing 100 mm glycine, and lysed in 50 mm sodium phosphate dibasic, 1 mm sodium pyrophosphate, 20 mm sodium fluoride, 2 mm EDTA, 2 mm EGTA, 1% Triton X‐100, 0.5 mm DTT, and complete protease inhibitor cocktail (Roche, Basel, Switzerland) lysis buffer. A portion of the whole cell lysate was kept as an input control. Remaining lysate was incubated with NeutrAvidin agarose beads (Thermo Fisher Scientific) for 3 h with slow rotation prior to washing three times with lysis buffer. Samples were boiled in loading buffer containing DTT prior to SDS/PAGE and western blot. Antibodies used were as follows: goat anti‐Galectin 3 (AF1197, R&D Systems, Minneapolis, MN, USA) diluted 1 : 10 000 and rabbit anti‐Vinculin (#4650, Cell Signaling Technology, Danvers, MA, USA) diluted 1 : 1000.

### Immunocytochemistry for Lgals3

*Lgals3*‐R200S wild‐type and mutant MEFs were harvested from e13.5 mouse embryos and grown directly on poly‐l‐lysine (2 μg·mL^−1^) coated glass coverslips placed in six‐well plates for 24 h prior to permeabilization and fixation with methanol for 15 min. Following three PBS rinses, cells were permeabilized with 0.1% Triton X‐100 in PBS for 5 min, followed by three more PBS rinses, prior to blocking in 1% BSA/5% rat serum for 30 min. Coverslips were then incubated in anti‐Gal3 antibody (R&D Systems, AF1197) diluted 1 : 1000 in 1%BSA/5% rat serum in PBS for 1–2 h, rinsed three times in PBS, incubated in secondary antibody conjugated to Alexa594 for 1 h, prior to three PBS rinses. DAPI (diluted 1 : 1000 in 1%BSA/PBS) was added to coverslips for 5 min and washed with 3× PBS and 1× water, before inverting onto a glass slide coated in mounting media. Optical imaging of cells was performed on a Nikon A1 + RSi laser scanning confocal microscope, equipped with DU4 high‐sensitivity detectors. Images were captured using Nikon NIS‐Elements AR 5.21.03 software with a 40× magnification plan fluor oil objective (NA 1.3), 402, 488 and 561 nm solid‐state laser lines, and 450/50, 525/50, and 595/50 nm bandpass emission filters. Intensity of the immunocytochemical staining was quantified using fiji software (National Institutes of Health, Bethesda, MD, USA). Briefly, cells were selected manually, and their mean and median fluorescence was automatically determined per unit area. We analyzed one biological sample per genotype and five slides per genotype were evaluated, with a total of *n =* 31 WT MEF cells and *n* = 40 *Lgals3*‐R200S mutant MEF cells. These data are presented as the mean ± the standard error of the mean where the error bars show the error between slides.

### Dual emission X‐ray absorptiometry (DEXA)

At 36 weeks of age, mice were anesthetized via inhalation of 2% isoflurane (TW Medical Veterinary Supply, Austin, TX, USA) with oxygen (1.0 L·min^−1^), weighed, and placed on a specimen tray in a PIXImus II bone densitometer (GE Lunar, Madison, WI, USA) for analysis. Bone mineral density (BMD) and body fat percentage were calculated by the PIXImus software based on the bone and tissue areas in the subcranial region within the total body image.

### Plasma isolation and enzyme‐linked immunosorbent assays (ELISA)

Mice were euthanized at 36 weeks for all following experiments. Immediately following euthanasia, approximately 0.5 mL of whole blood was collected by heart puncture and transferred to a microcentrifuge tube containing 5 μL of 0.5 m ethylenediaminetetraacetic acid (EDTA) pH 8.0. To separate plasma, tubes were centrifuged at 6000 ***g*** for 6 min. Plasma Gal‐3 from male and female wild‐type and heterozygous mice was measured using a DuoSet ELISA Development System kit (D1197; R&D Systems). Wells were coated with 100 μL of 2 μg·mL^−1^ Gal‐3 capture antibody. 1 μL of plasma was added per well. Recombinant Gal‐3 was used to generate a dilution curve for quantification.

### Micro‐computed tomography (μCT)

Right lower limbs and spines were defleshed and fixed in 10% neutral buffered formalin (NBF) for 72 h, rinsed with sterile distilled water, and stored in 70% ethanol at 4 °C. Whole femurs and L3 vertebrae were imaged using a desktop SkyScan 1172 microCT imaging system (SkyScan, Kontich, Germany). Scans were acquired at 80 kV using a 5.98 μm voxel size. The femoral trabecular volume encompassed regions 0.25–1.75 mm from the distal growth plate. For analyses of trabeculae within the body of L3 vertebrae, a 1.5 mm volume centered on the midpoint was used. Cortical measurements were obtained from a 0.6 mm segment that was 45% of the distance proximal of the length of the diaphysis from the growth plate (diaphysis length = distance of femoral head‐distance of the growth plate). Tissue mineral density and bone mineral density values were obtained using a standard regression line generated by converting the attenuation coefficients to mineral density from scans of hydroxyapatite standards with known densities (0.25 and 0.75 g·cm^−3^).

### Mechanical testing

Left femurs were defleshed, wrapped in PBS pH 7.2 soaked gauze, and stored at −20 °C. To calculate tissue‐level mechanical parameters, femurs were thawed at room temperature for 30 min in PBS pH 7.2 and analyzed by μCT at 80 kV with a 9.98 μm voxel size. Analyses were performed as described for cortical bone measurements for the right femurs—prior to −20 °C storage. For mechanical testing, femurs were thawed and equilibrated to room temperature for 2 h in PBS pH 7.2. Following equilibration, a standard four‐point bend test was performed using a TestResources 570L axial‐torsional screw‐driven testing system (TestResources, Shakopee, MN, USA) with a displacement rate of 0.005 mm·s^−1^. The distances between the lower and upper supports were 7.3 and 3.5 mm, respectively. The supports had radii of curvature of 0.5 mm at each point of contact with the femur. Displacement was applied by the upper supports in the anterior–posterior direction such that the anterior of the femur was in compression and the posterior was in tension. Force and displacement were directly measured from the load cell and crosshead, respectively. Tissue level mechanical parameters (max stress and elastic modulus) were calculated as described for four‐point bending [35] where max stress = (max force*a*c)/(2*I*_min_) and elastic modulus = stiffness*(*a*2/(12*I*_min_))*(3*L*−4*a*); where *a* = the distance between an upper and lower support beam, *L* is the distance between the lower support beams, *I*_min_ is the minimum calculated value of inertia, and c is the radius of the bone. *I*_min_ and c were obtained by μCT.

### Statistical analyses

A χ^2^ test (df = 5; α = 0.05) verified Mendelian distribution of pups born from *Lgals3*‐R200S heterozygous crosses. For most comparisons, the differences between *Lgals3*‐wild‐type (+/+) and *Lgals3*‐R200S (KI/+ and KI/KI) mice were determined using two‐way ANOVAs within age groups (sex, genotype). The Holm‐Sidak method was used in *post hoc* analyses to identify significant differences (α = 0.05). However, due to the large difference in plasma Gal‐3 levels between male and female mice, one‐way ANOVAs with Dunnett's *post hoc* tests were performed within sex between the genotypes.

## Results

### Generation of *Lgals3‐*R200S allele using CRISPR/Cas9

**Fig. 1:**
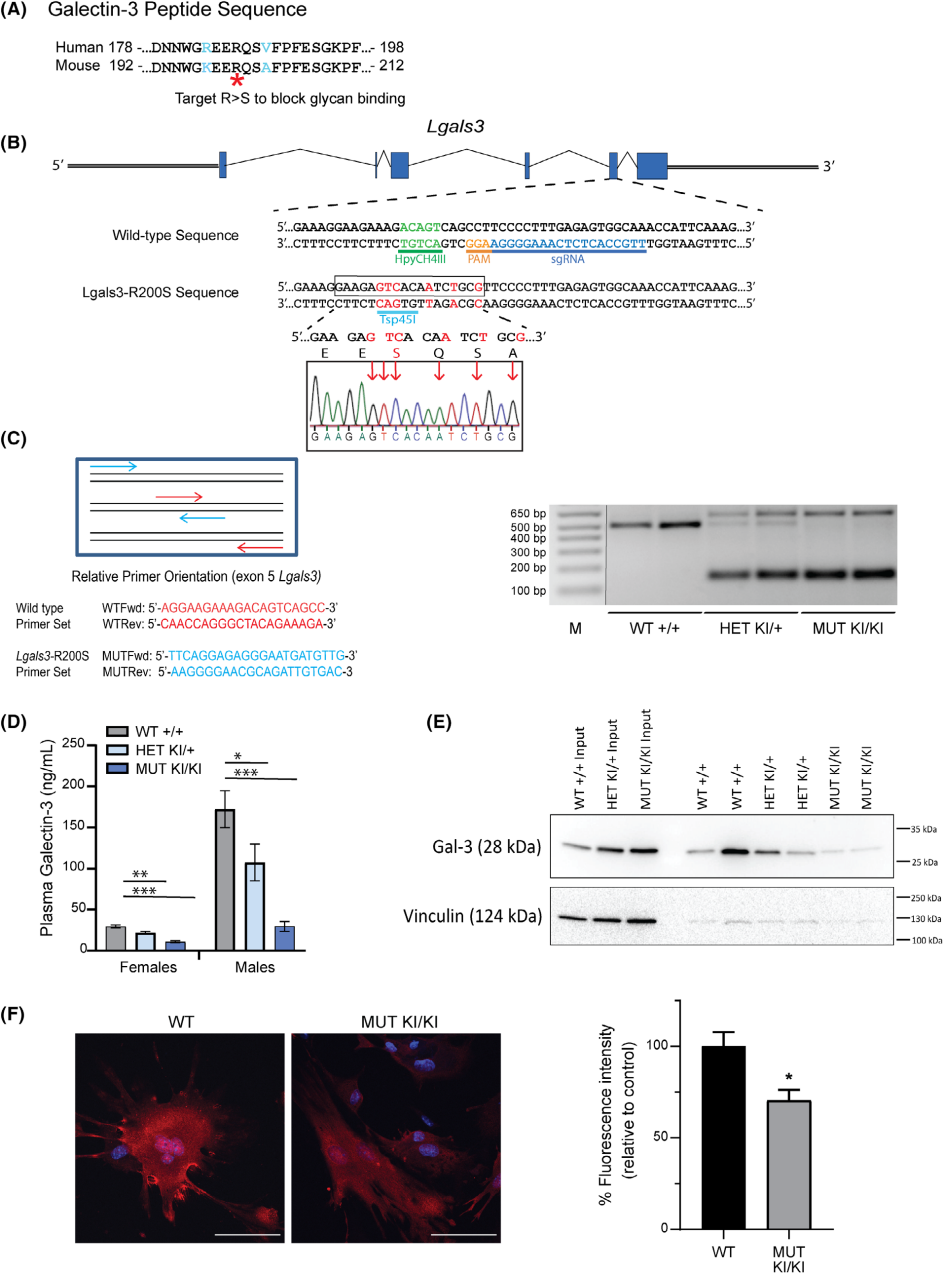
Generation of *Lgals3*‐R200S knock‐in allele by CRISPR/Cas9. (A) Comparison of human and mouse Gal‐3 amino acid sequence and identification of an essential arginine to mutate to generate the glycan‐binding deficient Gal‐3. (B) The target sequence of the *Lgals3* gene showing PAM and sgRNA binding site. Red letters indicate nucleotides to be mutated following homologous recombination of the single‐stranded oligonucleotide (ssODN) template. Sanger sequencing confirmation of incorporation of mutations. (C) PCR mediated identification of mice with wild‐type and *Lgals3*‐R200S alleles. M, molecular weight ladder; MUT, mutant; WT, wild‐type. Expected amplified product sizes are 483 bp for wild‐type and 131 and 586 bp for the R200S knock‐in allele. (D) Plasma serum levels of Gal‐3 in 36‐week‐old WT (+/+) female *n* = 10 and male *n* = 12, HET (KI/+) female *n* = 15 and male *n* = 11, and MUT (KI/KI) female *n* = 15 and male *n* = 13 animals. The samples were run in duplicate and the assay was run once. Values are expressed as mean ± SEM (*n* = 9–15). Dunnett's *post hoc* analysis adjusted *P*‐values compared to wild‐type; bold values highlight *P < 0.05, **P < 0.01, ***P < 0.001. (E) Immunoblot of Gal‐3 and vinculin on the cell surface of mouse embryonic cells isolated from WT (+/+), HET (KI/+), and MUT (KI/KI) embryos. Two biological replicates were evaluated per genotype and the assay was run once. (F) Gal‐3 immunocytochemistry of permeabilized MEFs from *Lgals3‐*R200S^KI/KI^ and WT mice. Gal‐3 is present throughout the cytosol and nucleus in both conditions. Gal‐3 protein levels are overall reduced by approximately 30%. Scale bar = 100 μm. One biological sample per genotype and five technical slide replicates per genotype were evaluated, with a total of *n =* 31 WT MEF cells and *n* = 40 *Lgals3*‐R200S mutant MEF cells. Quantification of Gal‐3 staining intensity is indicated on the right, data is mean ± SEM. Asterisks indicate a significant difference, *P < 0.05.

We mutated the homologous arginine to serine in the mouse *Lgals3* gene using CRISPR/Cas9 (*Lgals3*‐R200S; Fig. 1A) to generate a version of Gal‐3 that retains some intracellular functions but is deficient in glycoprotein binding, both extracellularly and to accumulate around disrupted vesicles intracellularly [36, 37, 38]. We identified the closest potential gRNA binding site with low predicted off‐target binding sites with a protospacer adjacent motif (PAM) that was within 8 bp of codon 200 in *Lgals3*. To introduce our mutation via homologous recombination, we designed a 200 bp single‐stranded oligo donor (ssODN) template by substituting the arginine codon (AGA) with a serine (TCA). To identify the *Lgals3‐*R200S allele easier by PCR, we included additional silent mutations that disrupted a unique HpyCH4III restriction site and generated a novel Tsp45I restriction site (Fig. 1B). We identified three out of 120 pups that were heterozygous for our targeted mutation by allele‐specific PCR of tail clip DNA from weanlings. These mice (one female and two males) were mated with wild‐type C57BL/6J mice to assess for germline transmission. Only one of the males was confirmed to carry the mutant allele in his germline and produced offspring with ~ 50% of the pups showing positive PCR results for the *Lgals3‐*R200S allele. Sanger sequencing revealed that these pups had the desired mutant allele (Fig. 1B). In this study, heterozygous offspring from this F1 generation were crossed to generate the mice analyzed for skeletal phenotypes. PCR genotyping was used to identify wild‐type (*Lgals3* ^+/+^), heterozygous (*Lgals3*‐R200S^KI/+^), and homozygous (*Lgals3*‐R200S^KI/KI^) mutant mice (Fig. 1C). Consistent with a reduction in Gal‐3 secretion, we observed significantly reduced Gal‐3 protein levels in the plasma of adult heterozygous and homozygous mutant mice (Fig. 1D).

We generated mouse embryonic fibroblasts (MEFs) to look at cell‐surface proteins, to confirm reduction of extracellular Gal‐3 protein in *Lgals3‐*R200S cells. Cell surface proteins were biotinylated and captured with NeutrAvidin Agarose. Western blot analysis showed cell surface Gal‐3 levels were decreased in *Lgals3‐*R200S^KI/+^ and *Lgals3‐*R200S^KI/KI^ cells compared to wild‐type (Fig. 1E). The absence of the cytoplasmic protein, vinculin, from the pull‐down lanes confirmed that the experiments worked to preferentially pull‐down biotinylated cell surface proteins. Immunocytochemistry of MEFs from *Lgals3‐*R200S^KI/KI^ mice confirmed that Gal‐3 is present in the cytosol and nucleus (Fig. 1F). Quantification of the mean and median amount of fluorescence per unit area indicated that Galectin‐3 both on the cell surface and inside the cell was reduced, by approximately 30% and 26% respectively, in *Lgals3‐*R200S^KI/KI^ MEFs (*P* = 0.024). From these studies, we conclude that the R200S mutation in mice reduced cell surface Gal‐3 and may have contributed to lower intracellular Gal‐3 levels. Further studies will be necessary to determine if the 25–30% reduction in intracellular Gal‐3 is biologically significant.

### No major changes in body composition in aged *Lgals3*‐R200S mice

The observed ratios of wild‐type, heterozygous and homozygous mice were observed in expected Mendelian ratios as determined by a χ^2^ test (data not shown). Like *Lgals3*‐KO mice [39], *Lgals3*‐R200S mice were grossly normal. Given that we previously observed significant bone phenotypes in Gal‐3 knockout animals at 36 weeks [5], we chose this timepoint to analyze the *Lgals3‐*R200S^KIKI^ model. Since we are phenotyping aging bones, we are unable to give insight into how this mutation impacts early bone formation. Analysis of body composition changes by dual‐energy X‐ray absorptiometry (DEXA) revealed no statistically significant differences for male or female *Lgals3‐*R200S mice in weight, areal bone mineral density (aBMD), or body fat percentage (%BF) as shown in Table 1.

**Table 1:** Body composition and plasma Galectin‐3 levels of wild‐type (*Lgals3* ^+/+^), heterozygous (*Lgals3‐*R200S^KI/+^), and mutant (*Lgals3*‐R200S^KI/KI^) mice^a^. aBMD, areal bone mineral density.

Variables compared	Females (36 weeks)	Males (36 weeks)
Bodyweight (g)		
Wild‐type	31.26 ± 1.60	44.41 ± 1.64
Heterozygotes	34.15 ± 1.41	46.86 ± 1.74
Mutant	34.32 ± 1.22	44.13 ± 2.07
Body fat (%)		
Wild‐type	32.19 ± 2.57	36.60 ± 1.94
Heterozygotes	36.26 ± 2.21	35.65 ± 1.51
Mutant	36.47 ± 2.40	31.57 ± 1.70
aBMD (g·cm^−2^)		
Wild‐type	0.057 ± 0.001	0.059 ± 0.001
Heterozygotes	0.057 ± 0.001	0.060 ± 0.001
Mutant	0.058 ± 0.001	0.059 ± 0.001

^a^ Values are expressed as mean ± SEM (*n* = 9–15). Dunnett's *post hoc* analysis adjusted *P*‐values compared with wild‐type; bold values highlight *P < 0.05, **P < 0.01, ***P < 0.001.

### Improved trabecular bone parameters in aged *Lgals3*‐R200S female mice

**Fig. 2:**
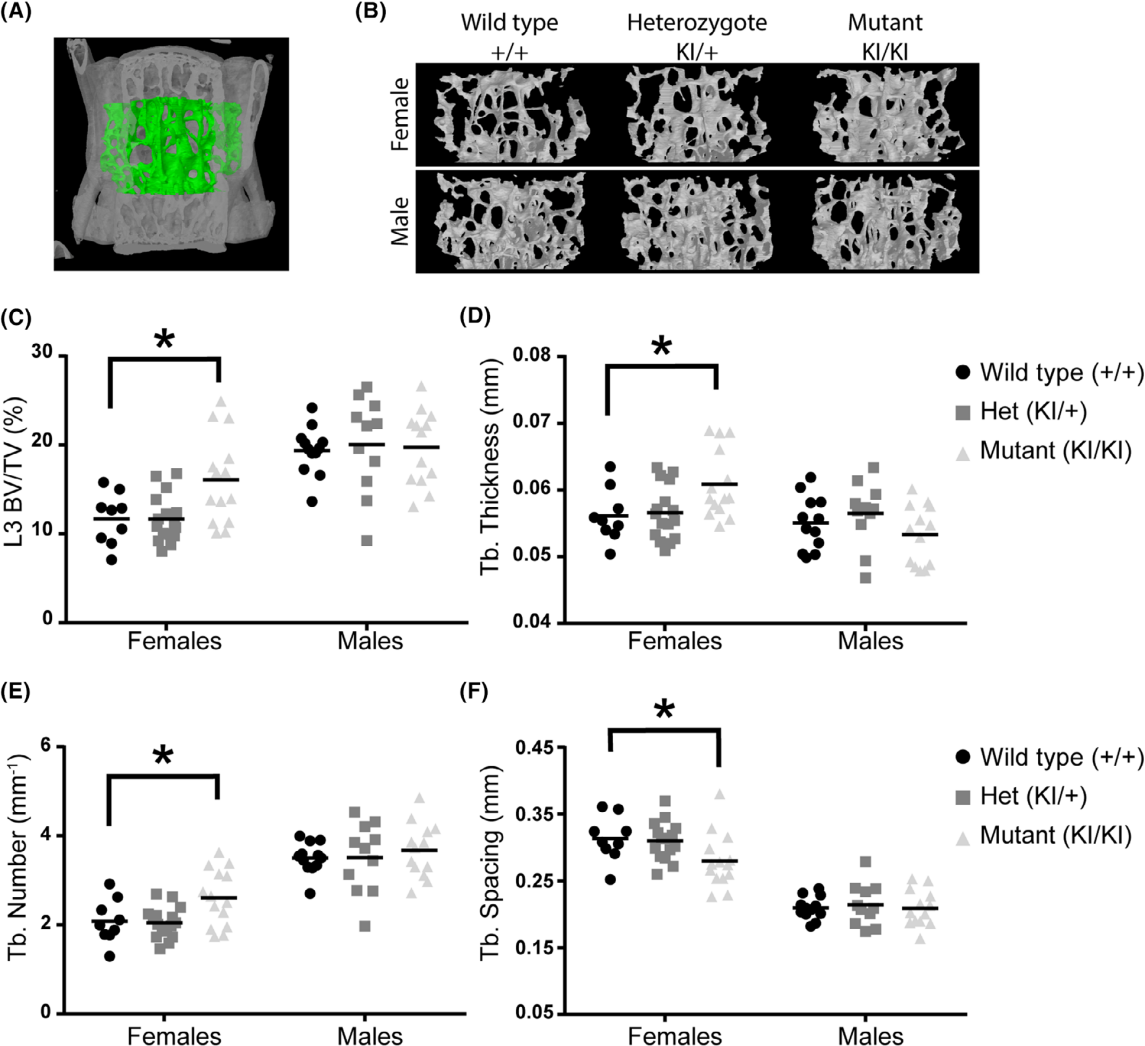
Enhanced trabecular bone mass at 36 weeks in *Lgals3‐*R200S females. (A) Volume of interest for L3 trabecular bone measurements, as assessed by μCT. (B) Representative trabecular bone volumes of mice in each of the treatment groups. Images were selected based upon median bone volume fraction within each sex:genotype. Scatter plots for bone volume fraction (BV/TV; C), trabecular (Tb.) thickness (D), Tb. Number (E), and Tb. Spacing (F). The following number of samples were analyzed: Wildtype (+/+) female *n* = 9 and male *n* = 9, Het (KI/+) female *n* = 15 and male *n* = 11, and Mut (KI/KI) female *n* = 14 and male *n* = 13. Statistical significance determined after Holm‐Sidak *post‐hoc* analyses of 2‐way ANOVAs. Asterisks indicate a significant difference, *P < 0.05.

We previously observed that female mice with complete deletion of the *Lgals3* gene also had significantly greater bone mass in both the femurs and L3 vertebrae at 36 weeks [5]. As shown in Table 2 and Fig. 2, we observed an increase in cancellous bone mass in L3 vertebrae at 36 weeks in female *Lgals3‐*R200S^KIKI^ mice. We analyzed bone structure of the L3 vertebra in wild‐type, *Lgals3‐*R200S^KI+^, and *Lgals3‐*R200S^KIKI^ mice (Fig. 2A). Evaluating μCT images of trabecular bone volumes in mice (Fig. 2B), *Lgals3‐*R200S^KIKI^ mice appeared to have denser bone than heterozygous mutant or wild‐type animals (Fig. 2B). *Lgals3‐*R200S^KIKI^ females had significantly elevated bone mineral density (BMD; +46.2%), bone volume fraction (BV/TV; +37.6%; Fig. 2C), trabecular thickness (Tb.Th; +8.9%; Fig. 2D), and trabecular number (Tb.N; +23.8%; Fig. 2E) as well as decreased trabecular spacing (Tb.Sp; −9.7%; Fig. 2F) in L3 vertebrae. The effect sizes observed in *Lgals3‐*R200S^KIKI^ female L3 trabecular bone were similar to what we observed in *Lgals3* ^KOKO^ females [5], with albeit a smaller overall effect size than in *Lgals3* ^KOKO^ females. Only *Lgals3‐*R200S^KIKI^ males had a statistically significant effect with enhanced connectivity density in the distal femur trabecular parameters (Conn.D; +29.9%; Table 2).

**Table 2:** Trabecular parameters of wild‐type (*Lgals3* ^+/+^), heterozygous (*Lgals3‐*R200S^K*I*/+^), and mutant (*Lgals3‐*R200S^KI/KI^) mice^a^. BV/TV, bone volume/total volume; Conn.D, connectivity density; Tb.N, trabecular number; Tb.Sp, trabecular spacing; Tb.Th, trabecular thickness.

Variables compared	Females		Males	
	Femur	L3	Femur	L3
BMD (g·cm^−3^)				
Wild‐type	0.075 ± 0.010	0.133 ± 0.013	0.181 ± 0.009	0.213 ± 0.010
Heterozygote	0.065 ± 0.005	0.131 ± 0.007	0.206 ± 0.018	0.224 ± 0.017
Mutant	0.096 ± 0.012	0.186 ± 0.015*	0.210 ± 0.016	0.219 ± 0.015
BV/TV (%)				
Wild‐type	3.95 ± 0.58	^b^	14.67 ± 0.82	19.37 ± 0.78
Heterozygote	3.07 ± 0.29	^b^	16.59 ± 1.86	20.07 ± 1.62
Mutant	5.48 ± 0.91	^b^	16.69 ± 1.29	16.08 ± 1.32
Tb.Th (mm)				
Wild‐type	0.055 ± 0.002	^b^	0.061 ± 0.002	0.055 ± 0.001
Heterozygote	0.055 ± 0.002	^b^	0.062 ± 0.001	0.056 ± 0.001
Mutant	0.057 ± 0.002	^b^	0.061 ± 0.002	0.053 ± 0.001
Tb.Sp (mm)				
Wild‐type	0.408 ± 0.035	^b^	0.246 ± 0.007	0.210 ± 0.005
Heterozygote	0.435 ± 0.019	^b^	0.242 ± 0.014	0.214 ± 0.009
Mutant	0.383 ± 0.019	^b^	0.233 ± 0.010	0.209 ± 0.008
Tb.N (mm^−1^)				
Wild‐type	0.735 ± 0.110	^b^	2.407 ± 0.116	3.508 ± 0.100
Heterozygote	0.558 ± 0.045	^b^	2.627 ± 0.258	3.515 ± 0.236
Mutant	0.923 ± 0.131	^b^	2.693 ± 0.177	3.677 ± 0.169
Conn.D (mm^−3^)				
Wild‐type	33.36 ± 5.73	64.55 ± 8.39	97.74 ± 7.08	120.25 ± 4.90
Heterozygote	22.99 ± 2.64	54.12 ± 3.15	110.63 ± 13.55	124.36 ± 12.38
Mutant	41.23 ± 7.82	87.38 ± 9.45	126.94 ± 13.06*	139.49 ± 9.24
Bone length (mm)				
Wild‐type	13.33 ± 0.16	3.60 ± 0.05	13.06 ± 0.06	3.67 ± 0.04
Heterozygote	13.22 ± 0.08	3.66 ± 0.04	13.22 ± 0.11	3.76 ± 0.05
Mutant	13.23 ± 0.09	3.67 ± 0.04	13.06 ± 0.11	3.74 ± 0.04

^a^ Values are expressed as mean ± SEM (*n* = 9–15); Holm‐Sidak *post hoc* analysis adjusted *P*‐values compared with wild‐type; bold values highlight *P < 0.05.

^b^ Data are displayed in Fig. 2.

### Enhanced cortical bone expansion in aged *Lgals3*‐R200S male mice

**Fig. 3:**
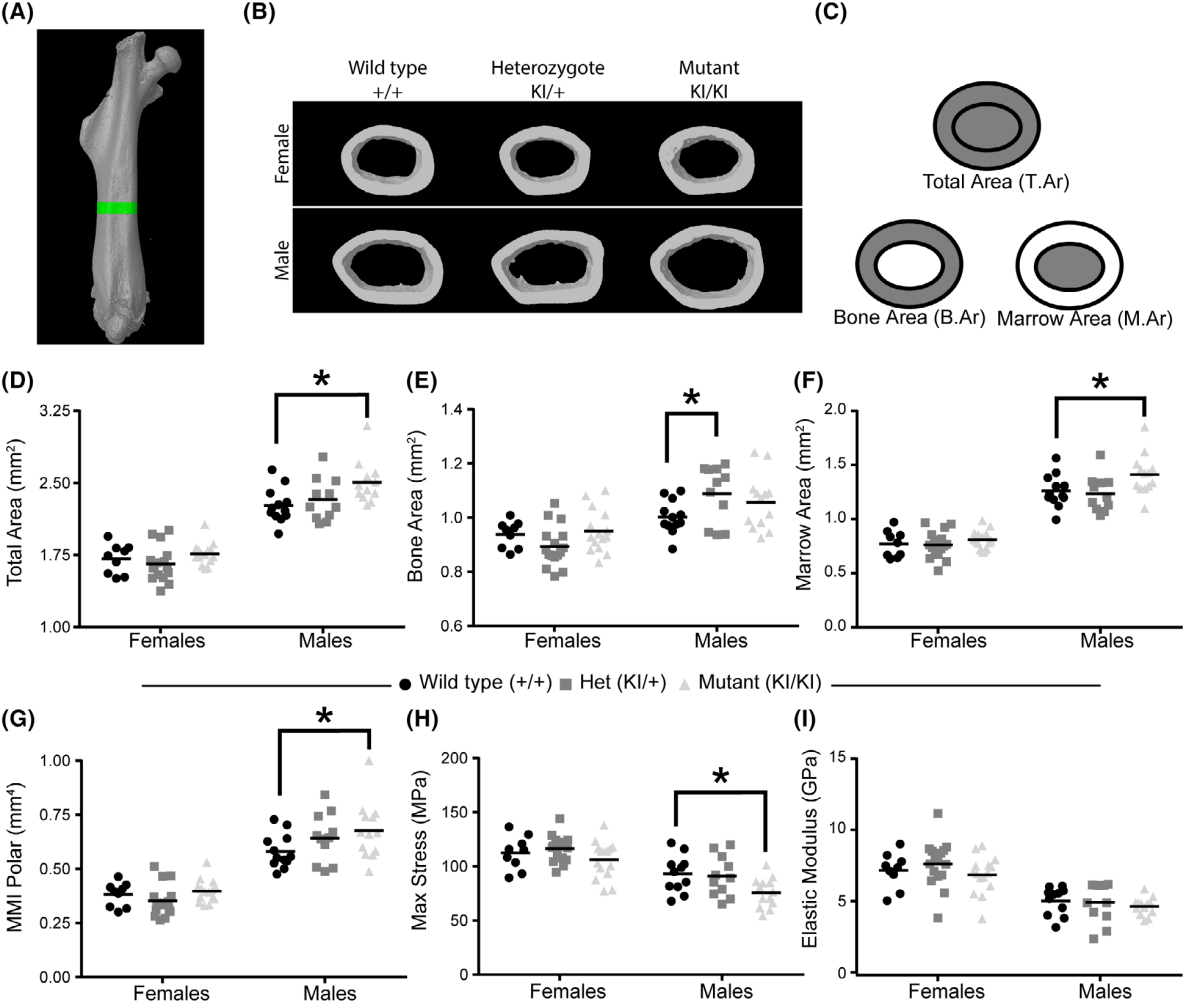
Enhanced cortical bone mass at 36 weeks in *Lgals3*‐R200S males. (A) Region of interest for cortical bone measurements, as assessed by μCT. (B) Representative cortical bone volumes of mice in each of the treatment groups. Images were selected based upon median Total Area measurements within each sex:genotype (C) Diagram showing areas represented by total area, bone area, and marrow area. Scatter plots from μCT data for total area (D), bone area (E), marrow area (F), and mean polar moment of inertia (MMI Polar; G). Scatterplots from derived tissue mechanical properties were measured by 4‐point bending, including max stress (H) and elastic modulus (I). The following number of samples were analyzed: Wildtype (+/+) female *n* = 9 and male *n* = 9, Het (KI/+) female *n* = 15 and male *n* = 11, and Mut (KI/KI) female *n* = 14 and male *n* = 13. Statistical significance determined after Holm‐Sidak *post hoc* analyses of 2‐way ANOVAs. Asterisks indicate a significant difference, *P < 0.05.

We previously observed a significant increase in cortical bone expansion by 36 weeks in *Lgals3*‐deficient mice, suggesting that loss of Gal‐3 protects bone from age‐related loss in mass [5]. Both male and female *Lgals3*‐deficient mice had an increase in total area (T.Ar), while female mice also had significantly increased bone area (B.Ar) [5]. In the current study, we took cortical bone measurements from 36‐week‐old mice using μCT to evaluate changes in cortical bone volume (Fig. 3A,B). We used μCT to determine T.Ar, B.Ar., and Marrow Area (M.Ar) (Fig. 3C). Notably, only male *Lgals3‐*R200S^KIKI^ mice had cortical expansion (Table 3), demonstrated by significant increases in T.Ar (+9.3%; Fig. 3D) and M.Ar; (+11.9%; Fig. 3F). The effect size of the increased T.Ar and M.Ar in *Lgals3‐*R200S^KIKI^ males was twice as strong compared with what we observed in *Lgals3* ^KOKO^ male mice [5]. *Lgals3‐*R200S^KI+^ males had a significant increase in B.Ar (+9%; Fig. 3E) compared with wild‐type males. The effect size in *Lgals3‐*R200S^KIKI^ females was roughly half of what we previously observed in *Lgals3* ^KOKO^ females at this age [5]. This data suggests that the extracellular function of Gal‐3 is not required in male mice for the protective effect on bone loss that occurs as a consequence of Gal‐3 loss but is required in female mice.

**Table 3:** Femoral cortical bone parameters of wild‐type (*Lgals3* ^+/+^), heterozygous (*Lgals3‐*R200S^KI/+^), and mutant (*Lgals3‐*R200S^KI/KI^) mice^a^. B.Ar, bone area; Ct.Th, cortical thickness; M.Ar, marrow area; MMI, mean moment of inertia; T.Ar, total area; TMD, tissue mineral density.

Variables compared	Females (36 weeks)			Males (36 weeks)		
	Wild‐type	Heterozygote	Mutant	Wild‐type	Heterozygote	Mutant
TMD (g·cm^−3^)	1.14 ± 0.02	1.09 ± 0.01	1.12 ± 0.02	1.06 ± 0.02	1.09 ± 0.02	1.10 ± 0.02
B.Ar/T.Ar (%)	55.1 ± 1.1	54.2 ± 0.9	54.0 ± 0.9	44.4 ± 0.8	47.2 ± 1.0	43.1 ± 0.8
Ct.Th (mm)	0.20 ± 0.01	0.21 ± 0.01	0.22 ± 0.01	0.19 ± 0.01	0.20 ± 0.01	0.19 ± 0.01
Porosity (%)	0.069 ± 0.041	0.058 ± 0.020	0.079 ± 0.029	0.129 ± 0.045	0.272 ± 0.167	0.316 ± 0.142
Pore thickness (mm)	0.016 ± 0.001	0.016 ± 0.001	0.019 ± 0.003	0.020 ± 0.003	0.028 ± 0.007	0.027 ± 0.004
Diameter (mm)	1.09 ± 0.01	1.07 ± 0.01	1.10 ± 0.01	1.13 ± 0.01	1.18 ± 0.02*	1.16 ± 0.02
MMI max (mm^4^)	0.24 ± 0.01	0.22 ± 0.01	0.25 ± 0.01	0.37 ± 0.01	0.42 ± 0.02	0.44 ± 0.02*
MMI min (mm^4^)	0.14 ± 0.01	0.13 ± 0.01	0.15 ± 0.01	0.21 ± 0.01	0.22 ± 0.01	0.24 ± 0.01*
Max force (N)	29.9 ± 1.7	30.2 ± 0.7	30.0 ± 1.2	35.1 ± 1.2	35.8 ± 2.1	32.3 ± 1.5
Stiffness (N·mm^−1^)	231.0 ± 10.7	232.1 ± 7.8	236.8 ± 8.7	240.6 ± 6.3	252.5 ± 16.4	261.1 ± 11.2

^a^ Values are expressed as mean ± SEM (*n* = 7–14); Holm‐Sidak *post‐hoc* analysis adjusted *P*‐values compared with wild‐type; bold values highlight *P < 0.05.

### Reduced bone quality in aged *Lgals3*‐R200S male mice

Our previous study suggested there was a decrease in tissue level strength (max stress) and stiffness (elastic modulus) in 36 week *Lgals3* ^KOKO^ male and female mice [5]. In the current study, male *Lgals3‐*R200S^KIKI^ mice, despite having significantly improved cortical geometry compared to wild‐type males (MMI min: +14%, MMI max: +19%), also had significantly reduced tissue level strength (Max stress: −18.6%; Fig. 3H) but did not have reduced whole bone strength (Max force). Female *Lgals3‐*R200S^KIKI^ mice, on the other hand, had no reduction in max force (Table 3) or max stress (Fig. 3H).

Stiffness results differed with what we observed in our earlier study (Table 3 and Fig. 3). Neither male nor female *Lgals3‐*R200S mice had a change in elastic modulus (Fig. 3I) or whole bone stiffness (Table 3).

## Discussion

In this study, we generated the *Lgals3*‐R200S allele using CRISPR/Cas9 and a single‐stranded DNA oligonucleotide as a template for homologous recombination. Mutation of the cognate arginine to serine in human Gal‐3 (R186S) prevents Gal‐3 secretion and glycan‐binding [30, 31, 40]. Because mutation of the functionally equivalent arginine in galectin‐7 also prevents glycan‐binding [32, 33], the R200S mutation in Gal‐3 should be functionally equivalent. As confirmation, our surface biotinylation experiment demonstrated a dose‐dependent reduction in surface Gal‐3 in heterozygous and homozygous *Lgals3*‐R200S mice. In our aged mouse bone studies, we observed a sex‐dependent increase in trabecular bone mass in female *Lgals3*‐R200S mice. Yet only male *Lgals3*‐R200S had significant increase in cortical bone expansion. The increased cortical bone expansion was coupled with reduced tissue quality (reduced max stress), and no change in tissue or whole bone stiffness values.

Similar to the findings presented here, we previously observed that female mice with genomic loss of Gal‐3 (*Lgals3*‐KO mice) had significant protection against age‐related trabecular bone loss between 24 and 36 weeks of age [5]. But *Lgals3*‐KO mice also had increased cortical bone expansion, whereas only male *Lgals3*‐R200S did in this study. The effect size of the increases in cortical bone size was greater in *Lgals3*‐KO females than males [5]. The similarities between *Lgals3*‐R200S and *Lgals3*‐KO mice (i.e., increased trabecular bone mass in female mice and increased cortical bone expansion in males) likely reflect the role of extracellular Gal‐3 loss in increasing bone mass. Conversely, the differences between the two models (tissue stiffness and lack of female cortical bone expansion) could reflect the role of intracellular Gal‐3.

The female dominance of the cortical bone expansion in *Lgals3*‐KO mice was further supported using a separate *Lgals3* null allele (*Lgals3*‐∆), where females once again had significantly increased trabecular and cortical bone mass at 36 weeks, but male *Lgals3*‐∆ had slight reductions in both cortical and trabecular bone mass. The apparent sex‐dependency of the bone phenotype was most likely due to diminished bone mass accrual in *Lgals3*‐KO males before 12 week of age [5, 41], which led us to speculate that *Lgals3*‐KO mice might have reduced androgen‐induced cortical bone expansion during puberty [42].

However, in *Lgals3*‐R200S mice, we observed a male dominant phenotype in cortical bone expansion. Our gonadectomy study suggested that global loss of Gal‐3 may lead to reduced bioavailability of androgens [6]. This would reduce the ability of androgen to support bone mass accrual during puberty [42]. Altered sex‐hormone regulation in the *Lgals3*‐KO mother during fetal development might also explain why a different skeletal phenotype (increased age‐related bone loss) has been reported when comparing *Lgals3*‐KO mice to litters of background matched wildtype‐mice [43]. Studies looking at systemic changes in hormones and growth factors in *Lgals3*‐KO and *Lgals3*‐R200S mice would help answer this question. In addition, conditional knockout of *Lgals3* at later developmental stages, such as pre‐ and postpuberty or in aged mice, and studies of pre‐ and postpubescent *Lgals3*‐R200S mouse cortical bone growth, will further clarify the timing of when extracellular Gal‐3 affects bone mass expansion.

## Conflict of interest

BOW has received a sponsored research award from Janssen Pharmaceuticals and is a member of the scientific advisory board and a shareholder of Surrozen. Neither of these are directly related to the work in this manuscript.

## Author contributions

Conceived of project idea: KAM & BOW; Generated Animals: KAM, CRD, & VVTC; Performed Experiments and Interpreted Data: KAM, CRD, PDS, & DD; Wrote Manuscript: KAM, CRD, PDS, & BOW.

## Data Availability

The data generated or analyzed during this study are included in this published article.
